# Oncogenic stress response mechanisms as new therapeutic targets in cancer treatment: A review

**DOI:** 10.1097/MD.0000000000042857

**Published:** 2025-06-13

**Authors:** Juan Iovanna, Matías Estaras, Daniel Grasso, Martin E. Fernández Zapico, Jose Luis Neira, Patricia Santofimia-Castaño

**Affiliations:** a Centre de Recherche en Cancérologie de Marseille (CRCM), INSERM U1068, CNRS UMR 7258, Aix-Marseille Université and Institut Paoli-Calmettes, Marseille, France; b Hospital de Alta Complejidad El Cruce, Florencio Varela, Argentina; c University Arturo Jauretche, Florencio Varela, Argentina; d Facultad de Farmacia y Bioquímica, Instituto de Estudios de la Inmunidad Humoral (IDEHU), Universidad de Buenos Aires, Buenos Aires, Argentina; e Division of Oncology Research, Schulze Center for Novel Therapeutics, Mayo Clinic, Rochester, MN; f Instituto de Biocomputación y Física de Sistemas Complejos, Unidad mixta GBsC-CSIC-BIFI, Zaragoza, Spain; g IDIBE, Universidad Miguel Hernández, Edificio Torregaitán, Elche, Spain.

**Keywords:** autophagy, DDR, HSP, NRF2-ARE, p53, redox-regulating proteins, stress granules, UPS

## Abstract

Activation of oncogenes, such as through mutations in Kirsten rat sarcoma viral oncogene homolog (KRAS), triggers profound disruptions in cellular homeostasis that set off a cascade of stress responses. These responses enable cells to cope with the array of challenges encountered during tumorigenesis by activating defense mechanisms that promote adaptation and survival. Key components of this oncogenic stress response include heat shock proteins, the ubiquitin-proteasome system, autophagy, nuclear factor erythroid 2-related factor 2–antioxidant response element signaling, DNA damage response proteins, p53, redox-regulating proteins, and stress granules. This review concentrates on KRAS-driven oncogenic transformation, as KRAS mutations are among the most common in human cancers, accounting for over 90% of pancreatic ductal adenocarcinoma cases, around 30% of lung cancers, and approximately 50% of colorectal cancers. We examine the intricate molecular interplay between oncogenic stress and the associated cellular defense mechanisms, emphasizing the key molecular events that follow KRAS activation. Importantly, the very pathways that allow cancer cells to adapt to oncogenic stress also offer novel therapeutic opportunities. By selectively targeting pivotal regulators within these stress response pathways, we can potentially disrupt the survival mechanisms of cancer cells. This strategy not only promises to enhance the effectiveness of existing treatments but also paves the way for the development of innovative therapies designed to combat tumor progression. In essence, exploiting oncogenic stress responses represents an original and promising therapeutic approach in the fight against cancer.

## 1. Introduction

### 1.1. Oncogene activation

Oncogenes, originally normal cellular genes known as proto-oncogenes, play crucial roles in maintaining cellular homeostasis^[1]^ and cellular processes such as cell growth and division. The transition from proto-oncogene to oncogene can involve several mechanisms, including point mutations, gene amplification, or chromosomal translocations.^[2]^ The conversion from proto-oncogene to oncogene profoundly alters gene function, leading to uncontrolled cell proliferation and suppression of apoptosis.^[3–5]^ The uncontrolled cell growth instigated by oncogenes can lead to the formation of tumors and contribute to the development of cancer. Understanding the dynamics of oncogene activation provides insights into the molecular underpinnings of cancer development. Targeting oncogenes and the pathways they affect represents a significant avenue for developing therapeutic strategies to curb uncontrolled cell growth and halt the progression of cancer.^[6,7]^

The typical example of a proto-oncogene is Kirsten rat sarcoma viral oncogene homolog (*Kras*), involved in signal transduction pathways that regulate cell growth.^[8]^ The *Kras* mutation is a common genetic alteration in various types of cancer and plays a key role in tumor initiation and progression. This mutation affects cellular signaling, leading to uncontrolled cell proliferation and tumor formation, especially in approximately 85% of pancreatic ductal adenocarcinomas (PDACs), 45% of colorectal adenocarcinomas and 30% of lung adenocarcinomas.^[9]^ Tumors with *Kras* mutations tend to be more aggressive and may be less responsive to certain treatments, including targeted therapies.^[10]^ Ongoing research aims to better understand these mutations and develop specific therapies to address tumors with mutated *Kras*, thereby improving treatment options for cancer patients.^[11]^

### 1.2. KRAS signaling pathway

The *KRAS* gene encodes a member of the Ras family of small GTPases, which regulate cellular signal transduction, orchestrating responses to external and internal cues. In its normal state, RAS genes, such as KRAS, regulate crucial cellular functions, including cell proliferation and differentiation. KRAS emerges as a key player frequently entangled in the complexities of oncogenic stress, since it is frequently activated in cancer. This pathway, involves mutations in *KRAS* gene, notably at the residues G12, G13, and Q61, that lock the Ras protein in its GTP-bound, thus inducing a constitutive activation of KRAS protein contributing to the dysregulation of cell proliferation, cell growth, cell survival, cell metabolism, cell motility and a host of gene transcriptional programs.^[12]^

One of the prominent downstream signaling pathways activated by KRAS is the rapidly accelerated fibrosarcoma-mitogen-activated protein kinase kinase-extracellular signal-regulated kinase pathway. Constitutively active KRAS protein stimulates rapidly accelerated fibrosarcoma kinases, setting off a cascade that culminates in the activation of extracellular signal-regulated kinase. This signaling cascade promotes cell cycle progression and cell differentiation and inhibits apoptosis. Dysregulation of the RAS pathway fuels the relentless growth of cancer cells, contributing to tumor development and progression; thus, understanding the intricacies of the RAS signaling pathway within the context of oncogenic stress provides different targets for therapeutic interventions.^[13]^

**Figure 1.**
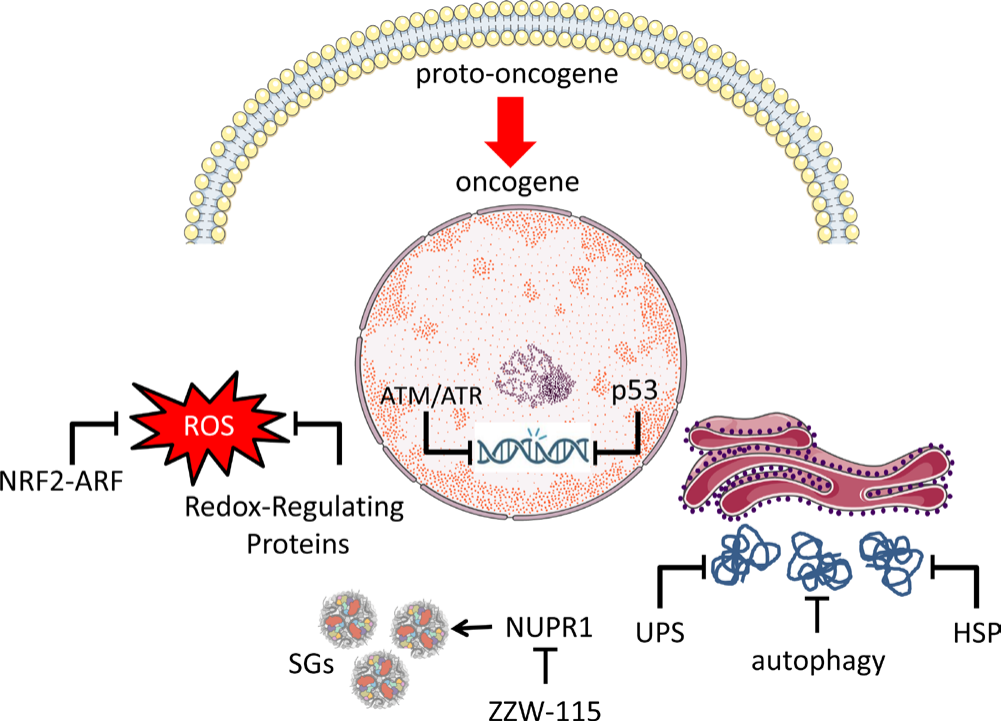
The main signaling cascades activated by mutated KRAS in PDAC. In a normal cell, the activation of the KRAS protein through binding of GTP and translocation to the plasma membrane is a tightly controlled process. However, in PDAC, the KRAS protein is often mutated at codons 12, 13, and 61 leading to the inactivation of its intrinsic GTPase activity resulting in constitutive activation of KRAS. Mutant KRAS can then promote tumorigenesis through multiple downstream signaling pathways. ARE = antioxidant response element, ATM = ataxia-telangiectasia mutated, ATR = ataxia-telangiectasia and Rad3-related kinase, HSPs = heat shock proteins, KRAS = Kirsten rat sarcoma viral oncogene homolog, NRF2 = nuclear factor erythroid 2-related factor 2, NUPR1 = nuclear protein 1, PDAC = pancreatic ductal adenocarcinomas, RTK = receptor tyrosine kinase, SGs = stress granules, UPS = ubiquitin-proteasome system.

The protein kinase B (AKT) pathway, also known as the PI3K/AKT/mammalian target of rapamycin signaling pathway, plays a pivotal role in cellular processes such as cell survival, proliferation, and metabolism. Upon activation, PI3K is activated, leading to the production of phosphatidylinositol (3,4,5)-trisphosphate. Phosphatidylinositol (3,4,5)-trisphosphate then recruits AKT to the cell membrane, where it is phosphorylated and activated. In the context of *Kras* mutation, the AKT pathway becomes intricately involved in the dysregulated signaling cascades involved in oncogenic transformation, associated with the promotion of cell survival and resistance to apoptosis.^[14,15]^ AKT phosphorylates and inactivates proapoptotic proteins, thereby inhibiting programmed cell death and allowing the survival of potentially damaged or transformed cells. In addition, activation of the PI3K/AKT pathway represses senescence induced by an activated RAS.^[16]^ Furthermore, by modulation of the AKT pathway, KRAS signaling regulates the activity of key proteins involved in cell cycle progression, such as cyclin-dependent kinases, contributing to uncontrolled cell division. Understanding the intricate interplay between *Kras* mutation and AKT pathway activation is crucial for developing targeted therapies aimed at disrupting these signaling cascades and, thereby, impeding the growth and survival of cancer cells. Ongoing research in this field continues to uncover potential therapeutic strategies to effectively target the AKT pathway in *Kras*-mutated cancers (see Fig. 1).

In addition to its involvement in the MAPK and PI3K/AKT/mammalian target of rapamycin pathways, KRAS regulates other signaling pathways that impact various cellular processes. For instance, KRAS plays a role in regulating the RAL guanine nucleotide dissociation stimulator pathway, where proteins such as Cdc42 act as effectors involved in cellular migration.^[17]^ Moreover, KRAS regulates pathways involving TIAM1 and RAC1,^[18]^ influencing cell shape, migration, adhesion, and actin cytoskeleton formation, among others. Furthermore, KRAS can also modulate the phosphatidylinositol signal pathway by activating PLC, which contributes to cellular signaling and regulation of various cellular functions.^[19]^ These additional pathways highlight the multifaceted role of KRAS in tumoral pathophysiology and underscore its significance in various cellular processes beyond the well-known and well-described MAPK and PI3K/AKT pathways.^[20]^

## 2. Oncogenic stress in cancer transformation

**Figure 2.**
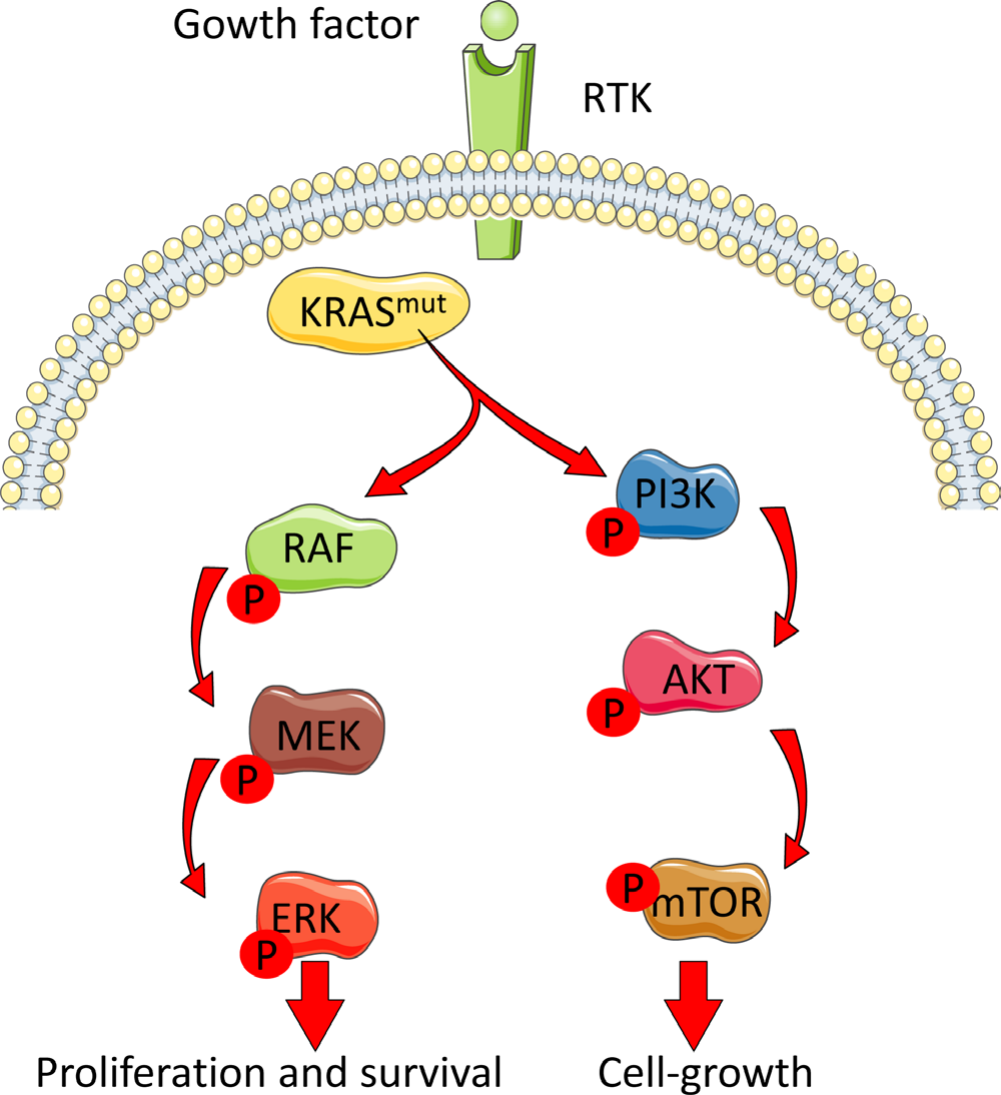
Simultaneous responses to oncogene-induced stress facilitate transformation following oncogenic activation. The intricate and numerous cellular crises triggered from oncogene activation to tumor formation offer new therapeutic opportunities. Identifying mechanisms such as the role of the IDP NUPR1 protein in stress granule formation during oncogenic crises is vital for discovering potential therapeutic targets. Understanding stress-associated proteins in the context of oncogenic stress is crucial for deciphering cellular responses that lead to survival or apoptosis. Targeting these pathways could provide cancer treatment opportunities by selectively inducing stress responses in cancer cells. Ongoing research continues to explore these molecular interactions for potential therapies. AKT = protein kinase B, ERK = extracellular signal-regulated kinase, IDP = intrinsically disordered protein, MEK = mitogen-activated protein kinase kinase, mTOR = mammalian target of rapamycin, NUPR1 = nuclear protein 1, RAF = rapidly accelerated fibrosarcoma, RTK = receptor tyrosine kinase.

Oncogenic stress refers to the cellular stress or damage caused by the abnormal activation of oncogenes. When oncogenes are inappropriately activated, they can disrupt normal cellular processes and lead to uncontrolled cell proliferation, a hallmark of cancer. This unregulated cell growth can induce various forms of stress on the affected cells, including DNA damage, metabolic stress, and imbalances in cellular signaling pathways. Oncogenic stress is an important concept in cancer biology because it is believed to contribute to the complex process of tumorigenesis. Under oncogenic stress, cells may activate protective responses or trigger apoptosis to prevent the propagation of damaged, potentially cancerous cells.^[21]^ Understanding the mechanisms of oncogenic stress is crucial for developing targeted therapies that aim to disrupt the specific pathways activated by oncogenes, ultimately inhibiting the growth and survival of cancer cells. Stress-associated proteins, also known as stress response proteins, play essential roles in cellular responses to various stressors, including oncogenic stress. These proteins help cells adapt to and survive under stress conditions. We will consecutively present the most important mechanisms developed by the cell in response to the transformation by oncogenes, namely heat shock proteins (HSPs), ubiquitin-proteasome system (UPS), autophagy, nuclear factor erythroid 2-related factor 2 (NRF2)-ARE, DNA damage response (DDR) proteins, p53, redox-regulating proteins, and stress granules in the context of oncogenic stress (see Fig. 2).

## 3. Heat shock proteins in the context of oncogenic stress

Oncogenic stress, marked by the abnormal activation and overexpression of oncoproteins, places significant demands on cellular homeostasis. Within this context, HSPs play a crucial role in mitigating the impact of oncogenic stress and facilitating cellular adaptation. HSP70 and HSP90, as major molecular chaperones, are at the forefront of managing the increased demand for protein folding during oncogenic stress.^[22]^ The overexpression of oncoproteins necessitates heightened chaperone function to ensure the proper folding of proteins crucial for cell growth and survival.^[23]^ HSP70 and HSP90 contribute to the stability and functionality of oncoproteins. However, they have a great versatility in their functions, since, for example, HSP70 participates in tumoral promotion by stimulating angiogenesis, suppressing cellular senescence, facilitating tumor cell metastasis,^[24]^ and acting as a biomarker for liquid biopsy; but it has also a dual role inducing anticancer response by activating immune cells.^[24]^ In addition, HSP27 acts as a molecular chaperone, preventing protein aggregation and ensuring proper folding; moreover, it acts as an antioxidant, inhibits apoptosis, and participates on the remodeling of the actin cytoskeletom.^[25,26]^ Its role is particularly significant during periods of oncogenic stress, where the cellular environment is challenged by the aberrant activation of oncoproteins. Furthermore, HSP60, predominantly localized in mitochondria, plays a vital role in folding mitochondrial proteins. Mitochondrial dysfunction is often associated with oncogenic stress, making the chaperone function of HSP60 crucial for maintaining mitochondrial integrity and cellular homeostasis.^[27]^ Additionally, HSP60 has also important roles by its capacity to interact with survivin,^[28]^ CypD,^[29]^ FHIT,^[30]^ and pro-caspase 3,^[31]^ exerting antiapoptotic and proapoptotic effects respectively. Cytosolic HSP60 interacts with MAPK family proteins,^[32]^ IKK,^[33]^ β-catenin,^[34]^ and p53^[35]^ to induce antiapoptotic, survival, and metastatic signaling extracellular HSP60 binds with membrane receptor TLR4 to trigger survival signals.^[36]^ HSP60 is distributed across the cell membrane interacting with integrin α3β1 to promote metastasis.^[37]^ In addition, this chaperone has been studied as a diagnostic and prognostic biomarker or therapeutic target.^[38]^ Dysregulation of these proteins can contribute to protein misfolding, aggregation, and cellular damage associated with the challenges posed by oncogenic transformation. Understanding how HSPs operate within the landscape of oncogenic stress provides valuable insights into the adaptive mechanisms that cancer cells employ for their survival and proliferation. Consequently, targeting these stress-associated proteins emerges as a promising strategy for disrupting the resilience of cancer cells under the influence of oncogenic stress, potentially opening new avenues for therapeutic interventions.^[23]^

## 4. Clinical significance of targeting oncogenic stress pathways dependent of HSPs

HSPs play a crucial role in cancer cell survival by stabilizing oncoproteins and preventing apoptosis. HSP70 is frequently overexpressed in various cancers, promoting tumor cell survival through apoptosis inhibition. Targeting HSP70 with inhibitors like VER-155008, which disrupts protein folding, has shown the potential in reducing tumor cell viability.^[39]^ Similarly, HSP90 is vital for the maintenance of multiple oncoproteins, and its inhibition with 17-AAG (Tanespimycin) leads to oncoprotein degradation, impairing cancer cell survival.^[40]^ Additionally, HSP27 and HSP60 contribute to tumor progression and therapy resistance, making their pharmacological inhibition a promising approach for overcoming resistance and improving treatment efficacy in cancer therapy.

## 5. Current challenges and future prospects

Despite their therapeutic promise, targeting HSPs presents several challenges. One of the main concerns is selectivity, as HSPs play essential roles in normal cellular function, and broad inhibition can lead to toxicity in noncancerous tissues. Additionally, tumor cells can develop resistance to HSP inhibitors by compensatory upregulation of other chaperones or activating alternative survival pathways.^[41]^ This limits the long-term effectiveness of single-agent HSP inhibitors. Furthermore, while several HSP inhibitors have demonstrated preclinical success, their clinical efficacy is often restricted due to poor bioavailability and off-target effects,^[42]^ making their translation into standard cancer therapy difficult. To overcome these challenges, current research is focused on developing combination therapies that pair HSP inhibitors with chemotherapy or immune checkpoint inhibitors to enhance their overall effectiveness.^[43]^ Additionally, selective targeting strategies are being explored to create inhibitors that preferentially target tumor-associated HSPs while sparing normal tissues.^[44]^ Personalized medicine approaches are also gaining attention, with efforts being directed toward identifying biomarkers that predict patient response to HSP-targeted therapies, allowing for more tailored treatment strategies. Several clinical trials are currently evaluating the efficacy of HSP-targeted therapies in combination with existing cancer treatments.^[45]^ Early findings suggest that these approaches could improve treatment response rates and reduce resistance, making them promising candidates for integration into standard cancer therapy. The continued refinement of these strategies, along with the development of novel, highly selective inhibitors, holds the potential to significantly impact cancer treatment outcomes in the coming years.

## 6. Ubiquitin-proteasome system in the context of oncogenic stress

Within the dynamic realm of cellular regulation, the UPS emerges as a crucial player, particularly when cells face with the complexities of oncogenic stress.^[46]^ This intricate system, designed for targeted protein degradation, takes on a heightened role in maintaining cellular equilibrium amid the challenges posed by aberrant oncoproteins. The UPS serves as a meticulous custodian of cellular health, orchestrating the degradation of misfolded or damaged proteins by tagging them with ubiquitin molecules, preventing the accumulation of potentially deleterious molecules. This ubiquitination serves as a molecular signal that designates proteins for dismantling by the proteasome, a formidable protein complex wielding proteolytic prowess. Oncogenic stress acts as a conductor signaling the cellular orchestra to activate the UPS.^[47]^ Modification on the UPS in major pathways such as β-catenin-TCF, NF-κB, tyrosine kinases, or TGFβ signaling also contributes to tumorigenesis.^[48]^ Understanding the nuanced responsiveness of the UPS to oncogenic stress unravels the sophisticated mechanisms cells employ to navigate the challenges posed by oncoproteins. The UPS, acting as a discerning gatekeeper, contributes significantly to the preservation of cellular homeostasis.

## 7. Clinical relevance of the UPS in cancer treatment

The UPS is crucial for protein degradation and regulates key oncogenic pathways contributing to tumor growth, metastasis, and therapy resistance. Proteasome inhibitors, including Bortezomib (Velcade), Carfilzomib (Kyprolis), and Ixazomib, have transformed multiple myeloma treatments by inducing apoptosis in cancer cells. Research is expanding their use in solid tumors,^[49]^ exploring combination strategies with chemotherapy and immunotherapy. Targeting UPS regulators, such as E3 ubiquitin ligases and deubiquitinases, offer novel cancer treatment approaches, while synthetic lethality strategies using UPS inhibition are being investigated in KRAS-driven cancers. Overall, UPS modulation represents a promising therapeutic strategy in both hematologic and solid tumors, with ongoing research focused on combination therapies, personalized approaches, and next-generation UPS-targeted drugs.

### 7.1 Current challenges and future prospects

Despite its therapeutic potential, targeting the UPS faces several challenges. One major limitation is the complexity of the system itself, as UPS components regulate numerous cellular processes beyond cancer, making selective targeting difficult without causing unintended toxicity.^[50]^ Additionally, tumor cells can develop resistance to proteasome inhibitors through compensatory mechanisms, including upregulation of alternative protein degradation pathways. Another challenge is the difficulty in designing small-molecule inhibitors that selectively target specific components of the UPS, such as E3 ubiquitin ligases or deubiquitinases, without affecting normal cellular processes. To address these challenges, researchers are developing more selective inhibitors and exploring combination therapies that enhance UPS-targeted treatments. Combining proteasome inhibitors with immunotherapies or chemotherapy has shown promise in overcoming resistance and improving treatment outcomes.^[51]^ Additionally, efforts to identify biomarkers that predict response to UPS-targeted therapies are underway, enabling a more personalized approach to treatment. Several clinical trials are ongoing to evaluate new UPS inhibitors, including next-generation proteasome inhibitors and novel molecules targeting specific UPS regulators, such as Marizomib^[52,53]^ and Oprozomib.^[54,55]^ Preliminary findings suggest that these approaches may improve therapeutic efficacy while minimizing side effects. As research progresses, further understanding of UPS regulation and its interplay with other cellular pathways will be essential for developing more effective and targeted cancer therapies.

## 8. Autophagy in the context of oncogenic stress

Among the intricate choreography of cellular responses, autophagy takes center stage as a pivotal process, particularly when cells confront the challenges of oncogenic stress. This cellular phenomenon, orchestrating the degradation and recycling of cellular components, stands as a dynamic guardian of cellular equilibrium. Autophagy, often referred to as the cell’s recycling system, plays a critical role in the disposal of damaged organelles and proteins. It involves the formation of autophagosomes, double-membraned vesicles that engulf cellular cargo slated for degradation. Under the duress of oncogenic stress, cells evoke the autophagic response as a survival strategy. The process is triggered to address the heightened demand for cellular maintenance, ensuring that damaged components, a common byproduct of oncogenic stress, are efficiently removed. Autophagy, induced under stress conditions, emerges as a sentinel maintaining cellular homeostasis during the tumultuous circumstances of oncogenic stress.^[56]^ By recycling cellular constituents, it facilitates the preservation of essential functions and prevents the accumulation of dysfunctional components that could compromise cellular integrity. The induction of autophagy in response to oncogenic stress underscores its adaptive role. Understanding the intricate interplay of autophagy within the context of oncogenic stress unveils a sophisticated cellular strategy for adaptation and survival.^[57]^ Autophagy, as a dynamic process, becomes a potential focal point for therapeutic interventions seeking to modulate cellular responses under the influence of oncogenic stress.

## 9. Clinical relevance of autophagy in cancer treatment

Autophagy plays a crucial role in cellular survival since it helps cancer cells adapt to oncogenic and metabolic stress and sustain tumor progression. However, excessive autophagy can also induce cell death, making it a potential therapeutic target. Modulating autophagy offers a dual approach in cancer therapy: inhibiting autophagy can sensitize tumor cells to chemotherapy and radiotherapy,^[58]^ while promoting autophagy in certain contexts can enhance tumor cell death.^[59]^ Targeting autophagic pathways represents a promising strategy to disrupt cancer cell survival and improve treatment outcomes.

### 9.1. Current challenges and future prospects

Despite the therapeutic promise of targeting autophagy, several challenges remain. One of the primary concerns is the dual role of autophagy in cancer, acting as both a prosurvival and a pro-death mechanism depending on the context.^[60]^ This complexity makes it difficult to determine when autophagy inhibition or activation would be the most beneficial strategy. Additionally, the development of resistance to autophagy-modulating therapies is a significant hurdle, as cancer cells can adapt their metabolic dependencies to circumvent the effects of autophagy inhibition or activation. Another major challenge is the identification of reliable biomarkers that can predict the response of tumors to autophagy-targeting therapies. Since different cancer types and even subpopulations of tumor cells may rely on autophagy to different extents, personalized treatment strategies are necessary. The heterogeneity of autophagic responses in various cancer types further complicates the development of universal therapeutic approaches. To address these challenges, ongoing research is focusing on combination therapies that integrate autophagy modulators with conventional chemotherapy, immunotherapy, or targeted therapies. Preclinical and early-phase clinical trials are investigating the potential of dual autophagy inhibition and checkpoint blockade in improving antitumor immune responses.^[61,62]^ Furthermore, advances in precision medicine and molecular profiling are aiding in the stratification of patients who are more likely to benefit from autophagy-targeting interventions. Several clinical trials are underway to evaluate the safety and efficacy of autophagy inhibitors such as hydroxychloroquine and chloroquine, particularly in combination with chemotherapy and radiation. Preliminary findings suggest that these combinations may enhance treatment responses in certain cancers, though further research is needed to establish standardized protocols.^[63]^ As the field advances, a deeper understanding of autophagy regulation and its interactions with other stress response pathways will be crucial for designing more effective therapeutic strategies.

## 10. NRF2-ARE pathway in the context of oncogenic stress

In the intricate tapestry of cellular responses, the NRF2 pathway emerges as a sentinel, particularly when cells confront the challenges of oncogenic stress.^[64]^ This signaling pathway, activated in response to oxidative stress, becomes a key player in orchestrating a cellular defense against the heightened oxidative milieu associated with oncogenic stress. The NRF2-ARE (antioxidant response element) pathway serves as a molecular conductor that composes the cellular response to oxidative stress. It is activated when the cellular environment is challenged by an excess of reactive oxygen species (ROS), which can result in oxidative damage to cellular components. Oncogenic stress, often accompanied by increased oxidative stress, triggers the activation of the NRF2-ARE pathway.^[65]^ The pathway response is finely tuned to neutralize the harmful effects of ROS generated during the aberrant activation and overexpression of oncoproteins. A pivotal outcome of NRF2 activation is the upregulation of antioxidant enzymes. These enzymes, including superoxide dismutase and catalase, act as molecular guardians, neutralizing ROS and preventing oxidative damage to cellular structures.^[66]^ Understanding the activation of the NRF2-ARE pathway within the context of oncogenic stress provides insights into the intricate mechanisms cells employ to counteract the oxidative challenges imposed by oncoproteins.^[67]^

## 11. Clinical relevance of the NRF2-ARE pathway in cancer treatment

The NRF2-ARE pathway plays a crucial role in cellular defense against oxidative stress, particularly under oncogenic stress conditions. Activated in response to elevated ROS, this pathway regulates antioxidant enzymes such as superoxide dismutase and catalase, neutralizing ROS and preventing oxidative damage to cellular structures. In cancer, NRF2 activation can confer resistance to oxidative stress, promoting tumor survival and therapy resistance. Understanding how NRF2 operates under oncogenic stress provides insights into potential therapeutic strategies targeting its regulatory mechanisms to enhance treatment efficacy in oxidative stress-driven cancers.

### 11.1. Current challenges and future prospects

Despite its importance in maintaining cellular homeostasis, the NRF2-ARE pathway presents several challenges as a therapeutic target. One of the primary difficulties is its dual role in cancer, while NRF2 activation protects normal cells from oxidative damage, its persistent activation in tumors can enhance survival, proliferation, and resistance to therapy. This complicates the development of NRF2-targeting drugs, as both inhibition and activation may be necessary depending on the cancer type and context.^[68]^ Another significant challenge is the complexity of NRF2 regulation, which involves multiple upstream regulators and crosstalk with other stress response pathways. The development of selective NRF2 modulators that specifically target tumor cells without affecting normal tissues remains a major hurdle. Additionally, NRF2-driven resistance to chemotherapy and radiotherapy necessitates combination approaches that counteract its protective effects in tumor cells while preserving its beneficial functions in normal tissues. Ongoing research is focused on identifying NRF2 inhibitors that can selectively suppress its activity in cancer cells, as well as combination strategies that enhance the effectiveness of chemotherapy and targeted therapies.^[69,70]^ Clinical trials evaluating NRF2 modulators, such as KEAP1 inhibitors and synthetic NRF2 repressors, are underway, with preliminary findings suggesting potential therapeutic benefits in oxidative stress-driven malignancies.^[71]^ Also, efforts to identify predictive biomarkers for NRF2-targeted therapies are crucial for patient stratification and personalized treatment approaches. As research advances, a deeper understanding of NRF2 regulation and its interactions with other oncogenic pathways will be essential for optimizing therapeutic strategies. Targeting NRF2 in combination with other stress response modulators, such as autophagy inhibitors or proteasome inhibitors, may offer a promising avenue for overcoming tumor resistance and improving treatment efficacy.

## 12. Redox-regulating proteins in the context of oncogenic stress

In the intricate realm of cellular equilibrium, redox-regulating proteins, exemplified by thioredoxin and glutathione, emerge as critical players, particularly when cells navigate the challenges of oncogenic stress.^[72]^ These proteins are instrumental in maintaining the delicate balance of cellular redox status. Their primary function is to manage the levels of ROS and maintain the intricate redox equilibrium essential for cellular functions. Oncogenic stress perturbs the redox status within cells. Redox-regulating proteins, in turn, respond to this imbalance induced by oncogenic stress, triggering adaptive mechanisms to restore redox homeostasis. Thioredoxin and glutathione, among others, engage in detoxifying ROS and rectifying oxidative damage caused by the stress imposed by oncoproteins. Thioredoxin acts as a thiol-disulfide oxidoreductase, participating in redox signaling and maintaining the reduced state of proteins and glutathione functions as a major antioxidant, neutralizing ROS and providing reducing equivalents for enzymes involved in detoxification.^[73]^ Interestingly, mutationally activated KRAS strikingly increased intracellular cystine levels and glutathione biosynthesis, whereas the SLC7A11/glutathione axis displays metabolic synthetic lethality with oncogenic KRAS.^[74]^ In addition, higher expression of GPX4, FTH1, and FTL, ferroptosis inhibitors proteins were also associated with poorer 5-year overall survival in patients with KRAS-mutant tumors in colorectal cancer.^[75]^ The engagement of redox-regulating proteins in response to oncogenic stress illustrates their adaptive role in restoring redox homeostasis.

## 13. Clinical relevance of ferroptosis induction in cancer treatment

Ferroptosis, an iron-dependent form of cell death driven by lipid peroxidation, has emerged as a promising therapeutic strategy in cancer. KRAS-mutant tumors exhibit increased reliance on antioxidant defenses to counteract oncogenic stress. Disrupting this metabolic dependency can sensitize tumor cells to ferroptosis, making it a viable approach for synthetic lethality in cancer treatment. The overexpression of ferroptosis inhibitors such as nuclear protein 1 (NUPR1)^[76]^ highlights the need for therapeutic interventions that restore susceptibility to oxidative stress-induced cell death.^[75]^ Targeting these regulators presents an opportunity to overcome therapy resistance, particularly in aggressive malignancies. Ferroptosis-based therapies, either alone or in combination with conventional treatments, hold potential for improving clinical outcomes in cancers with heightened redox dependencies.

### 13.1. Current challenges and future prospects

Despite the therapeutic potential of ferroptosis-based strategies, several challenges must be addressed. One major limitation is the variability in tumor dependence on redox homeostasis, which complicates patient selection for ferroptosis-targeting therapies. Furthermore, the identification of specific biomarkers to predict ferroptosis susceptibility remains a key research priority. Another challenge is the tumor’s ability to develop compensatory mechanisms that counteract ferroptosis induction, potentially leading to resistance over time. To overcome these hurdles, ongoing research is focusing on combination therapies that enhance ferroptosis induction while minimizing resistance. Strategies incorporating ferroptosis inducers with inhibitors of redox-regulating proteins, such as GPX4 inhibitors or glutathione synthesis blockers, are being explored.^[77]^ Also, preclinical studies are evaluating the integration of ferroptosis-targeting agents with conventional chemotherapy, immunotherapy, and radiation therapy to enhance treatment efficacy.^[78–80]^ Several clinical trials are currently assessing the safety and effectiveness of ferroptosis-inducing compounds such as Sorafenib in cancer treatment.^[81]^ Early findings suggest that these approaches may improve tumor response rates, particularly in cancers with heightened redox dependencies. As research progresses, refining ferroptosis-based strategies through patient stratification and personalized approaches will be essential for optimizing therapeutic outcomes.

## 14. DNA damage response proteins in the context of oncogenic stress

Within the intricate landscape of cellular safeguarding, DDR proteins emerge as vigilant guardians, especially when cells face with the challenges of oncogenic stress. These proteins play a pivotal role in sensing and repairing DNA damage induced by the tumultuous presence of oncoproteins. Oncogenic stress acts as a potent inducer of DNA damage, prompting a robust activation of DDR proteins.^[82]^ Two key players in the DDR orchestra are ataxia-telangiectasia mutated (ATM) and ATR (ataxia-telangiectasia and Rad3-related) kinases.^[83]^ These kinases act as molecular sentinels, detecting DNA damage and launching the appropriate repair pathways. Upon sensing DNA damage induced by oncogenic stress, ATM and ATR kinases initiate specific DNA repair pathways.^[84]^ ATM is primarily involved in responding to double-strand breaks, while ATR responds to single-strand breaks and other forms of DNA damage. Recently, several proteins have been identified as DDR proteins that promote the proliferation of KRAS-mutated cell by decreasing the accumulation of DNA lesions, such as VCP/p97, a pleiotropic protein regulator of the DDR,^[85]^ or CHK1, a key component of the ATR-CHK1 DDR pathway, both in PDAC.^[86]^ Furthermore, KRAS mutations lead to the activation of NRF2 antioxidant signaling to increase 53BP1 gene transcription, which plays an important role in nonhomologous end-joining repair.^[87]^ These pathways are crucial for maintaining genomic integrity. Understanding the role of DDR proteins within the context of oncogenic stress sheds light on the intricate mechanism’s cells employ to counteract the genomic challenges posed by oncoproteins. Targeting DDR pathways presents a promising avenue for therapeutic interventions aimed at enhancing DNA repair mechanisms and preserving genomic stability in the face of oncogenic stress.

## 15. Clinical relevance of DDR proteins in cancer treatment

DDR proteins serve as essential guardians of genomic integrity, particularly under oncogenic stress, where excessive DNA damage occurs due to the aberrant activation of oncoproteins. These proteins detect DNA lesions and initiate repair mechanisms to restore genomic stability. Key DDR components respond to double-strand and single-strand breaks orchestrating critical repair pathways. In KRAS-mutant cancers, DDR proteins contribute to tumor cell survival by minimizing DNA damage accumulation, while NRF2 activation further enhances DNA repair through upregulation of 53BP1,^[87]^ a key factor in nonhomologous end-joining repair. Targeting DDR pathways presents a promising therapeutic strategy by inhibiting DNA repair mechanisms, thereby increasing genomic instability and sensitizing tumors to treatment. DDR inhibitors are currently being explored as potential therapeutic agents to enhance the efficacy of DNA-damaging treatments in KRAS-driven cancers.

### 15.1. Current challenges and future prospects

Despite their therapeutic potential, DDR-targeting strategies face several challenges. One major obstacle is the redundancy and complexity of DNA repair pathways, which allows cancer cells to compensate when one pathway is inhibited. Moreover, many DDR inhibitors have shown promising preclinical results but have struggled with toxicity and off-target effects in clinical trials.^[88]^ Another significant challenge is the development of resistance mechanisms, where tumor cells adapt by upregulating alternative DNA repair pathways or modifying their dependency on DDR proteins. To address these limitations, ongoing research is focusing on combination therapies that integrate DDR inhibitors with chemotherapy, radiation therapy, and immune checkpoint blockade. These approaches aim to exploit synthetic lethality, where DDR inhibition enhances the efficacy of DNA-damaging treatments while selectively targeting tumor cells. Moreover, advances in precision medicine are helping to identify biomarkers that predict patient responses to DDR inhibitors, allowing for more tailored treatment strategies. Several clinical trials are currently evaluating the safety and effectiveness of DDR inhibitors in combination with other cancer therapies. Early results suggest that these approaches may improve treatment responses in DDR-deficient tumors,^[89–91]^ particularly those with mutations in KRAS, BRCA1/2, or ATM. As research continues, optimizing DDR-targeted therapies through patient stratification and novel drug development will be critical for improving clinical outcomes.

## 16. p53 in the context of oncogenic stress

Among the cellular responses triggered in the oncogenic process, p53 stands as a central figure. This tumor suppressor protein plays a pivotal role in regulating the cellular responses to stress, serving as a guardian of genomic integrity. p53 functions as a master regulator, overseeing cellular responses to various stresses.^[92]^ Its primary role is to safeguard the integrity of the genome and maintain cellular homeostasis. Oncogenic stress, marked by the aberrant activation and overexpression of oncoproteins, prompts the activation of p53.^[93]^ This activation is a strategic response to the genomic challenges imposed by oncoproteins. In the context of oncogenic stress, p53 can induce different cellular responses based on the severity of the stress: i/p53 can initiate cell cycle arrest, halting the progression of the cell cycle to allow for the repair of damaged DNA. This temporary pause serves as a protective mechanism against the propagation of damaged genetic material; ii/p53 facilitates the activation of DNA repair mechanisms, ensuring the restoration of genomic integrity. By promoting repair processes, p53 contributes to the maintenance of a stable and functional genome; iii/in cases of severe stress or irreparable DNA damage, p53 can trigger apoptosis. This drastic measure serves to eliminate cells with damaged DNA, preventing the propagation of potentially harmful genetic alterations; iv/p53 ability to dynamically regulate cellular fate in response to oncogenic stress underscores its significance as a guardian of cellular integrity. The multifaceted roles of p53 contribute to the preservation of genomic stability and the prevention of unchecked cellular proliferation associated with oncogenic stress.^[94]^ Understanding the role of p53 within the context of oncogenic stress provides insights into the sophisticated mechanisms cells employ to counteract the genomic challenges posed by oncoproteins. Leveraging p53’s regulatory capacities holds promise for therapeutic interventions aimed at controlling cellular responses under the influence of oncogenic stress.

## 17. Clinical relevance of p53 in cancer treatment

p53 plays a key role in maintaining cellular homeostasis by initiating cell cycle arrest to allow DNA repair, activating repair mechanisms to restore genomic stability, and inducing apoptosis in cases of irreparable DNA damage. In cancer, p53 function is often disrupted, leading to unchecked proliferation and genomic instability. Targeting p53 pathways offers a promising therapeutic approach by restoring its tumor-suppressive functions or leveraging its regulatory capacity to induce apoptosis in cancer cells. p53-based therapies, including small molecules that reactivate mutant p53 or enhance its signaling, are being explored as potential strategies for cancer treatment.

## 17.1. Current challenges and future prospects

Despite its critical role in tumor suppression, p53-targeted therapies face several challenges. One major issue is that p53 mutations are highly diverse, making it difficult to develop a universal therapeutic strategy. Some tumors harbor gain-of-function mutations that turn p53 into an oncogenic factor rather than a tumor suppressor, further complicating treatment approaches. Additionally, restoring wild-type p53 function in mutant cells remains a significant challenge due to difficulties in developing small molecules that can selectively target mutant p53 while sparing normal tissues. To overcome these challenges, research is focusing on novel approaches such as small molecules that stabilize or reactivate mutant p53, synthetic lethality strategies that exploit vulnerabilities in p53-deficient tumors, and immunotherapies that target p53-expressing cancer cells. Clinical trials evaluating p53 reactivators such as APR-246 (Eprenetapopt) are showing promise in hematologic and solid tumors. Furthermore, combination therapies integrating p53 reactivators with chemotherapy or immune checkpoint inhibitors are being explored to enhance treatment efficacy.^[95]^ As research advances, the development of biomarkers to identify patients who would benefit most from p53-targeted therapies will be crucial. Personalized medicine approaches, in conjunction with next-generation drugs, will likely play a pivotal role in optimizing p53-based cancer treatments.

## 18. Stress granules in the oncogenic context

Stress granules are cytoplasmic assemblies of RNA-binding proteins and translation factors that form under stress conditions. When the stress response is induced in suffering cells, different stress sensors are activated, coordinating the cellular adaptation to stress. Stress granule formation and dynamics could be considered as regulating, under stress conditions, mRNA localization, translation, and degradation, as well as signaling pathways.^[96]^ Stress granule formation has major advantages for cell physiology since it minimizes energy expenditure, controls proteo- and ribo-stasis, and improves cell survival under damaging conditions.^[97]^ Moreover, the stress granule assembly is highly dynamic, with quick assembly under stress and rapid dispersion upon stress removal.^[98]^ Stress granules are classified within a newly recognized group of membraneless organelles, hypothesized to consist of multicomponent, viscous liquid condensates that emerge spontaneously through liquid-liquid phase separation (LLPS).^[99]^ An LLPS occurs when a molecule, or mixture of molecules, forms a network of multivalent weak interactions, which allows those molecules to concentrate into a separate phase. In vitro, LLPS-derived droplets exhibit several key characteristics observed in stress granules within cells, such as the ability to fuse, wet surfaces, undergo shear deformation, and maintain dynamic behavior. LLPS can be initiated in vitro by elevated local concentrations of proteins that contain intrinsically disordered regions (IDRs). Given that stress granules are rich in proteins harboring IDRs, it has been proposed that these disordered regions in RNA-binding proteins mediate both heterotypic and homotypic interactions that promote the nucleation of stress granules. This hypothesis is reinforced by in vitro findings showing that high concentrations of IDRs can independently drive LLPS, likely via a combination of weak electrostatic and hydrophobic interactions between similar and dissimilar protein partners. Studies of mammalian stress granules indicate they possess a biphasic structure: an outer, dilute shell that dissipates upon cell lysis, consistent with an LLPS-dependent assembly stabilized by weak interactions, and a more persistent internal core. Notably, IDR-driven liquid droplets formed in vitro have been observed to transition into a secondary, less dynamic state containing stable, amyloid-like assemblies. A plausible and streamlined model suggests that stress granules initially arise through LLPS, driven by multivalent, transient, and weak interactions. With continued supersaturation of IDRs, this initial liquid phase progressively transitions into a denser, less dynamic state, giving rise to the stable core structures of stress granules. Consequently, targeting and disrupting the IDR-mediated interactions responsible for LLPS and stress granule formation presents a potential therapeutic approach to compromise the viability of cancer cells.

Stress granules are considered membraneless RNA-protein condensates that form under conditions of impaired translation initiation and exhibit a biphasic organization, comprising a stable internal core surrounded by a more dilute peripheral shell. The precise temporal sequence governing the assembly and disassembly of these structural components is still unclear. However, kinetic studies indicate that core formation occurs early during granule assembly. The disassembly process appears to be sequential, with the outer shell dispersing first, followed by the resolution of the core. Notably, experimental manipulations that affect LLPS driven by IDRs of RNA-binding proteins in vitro tend to exert inverse effects on stress granule assembly within living cells.^[100]^ Taken together, these observations argue that stress granules assemble through a multistep process initiated by stable assembly of untranslated membraneless RNA-protein into core structures, which could provide sufficient high local concentrations to allow for a localized LLPS driven by IDRs on RNA-binding proteins.^[101]^

The involvement of stress granules in cancer initiation and progression is an emerging concept in tumor biology.^[102]^ In this regard, responding and adapting to stress is important both in the development and progression of the cancer, and in its resistance to anticancer therapies.^[103]^ Interestingly, recent studies have indicated that stress granules participate in cancer development and metastasis, making them an attractive target for new cancer therapies.^[104]^ In PDAC, stress granule formation has been recently proposed to function as a resistance mechanism to current chemotherapies,^[105]^ thus implying that interfering with stress granule formation could provide an effective approach to sensitizing tumors to chemotherapeutic agents. Moreover, stress granules are markedly elevated in mutant *Kras* cells where they probably function as a resistance mechanism to current chemotherapies.^[106]^ Targeting essential proteins that drive stress granule formation could therefore provide an effective approach to sensitizing *Kras* tumors to chemotherapeutic agents. The key functions of the stress granules concerns to (i) the storage and protection of the mRNA and their associated proteins during stress conditions; (ii) the cellular adaptation since the formation of stress granules allows cells to reprogram their gene expression profile, promoting the translation of stress-responsive proteins while inhibiting the translation of nonessential proteins; (iii) the response to various stressors, such as oncogenic stress, among others, to help the cell adapt and survive; and (iv) assembling and disassembling of the granules, depending on the severity and duration of the stress. In summary, stress granules are dynamic cellular structures that form in response to stress conditions, serving as a protective mechanism to reorganize cellular processes and promote cell survival during challenging environments.^[102]^ The proteins involved in stress granule formation and regulation play key roles in orchestrating the cellular stress response.

## 19. Clinical relevance of stress granules

*Kras* mutations, prevalent in many human tumors, notably PDAC, colorectal, and lung cancers,^[107]^ induce a strong constitutive oncogenic activation and its cellular stress response. Current strategies target mutant RAS proteins either directly^[108]^ or by identifying vulnerabilities in oncogene-addicted tumor cells.^[109,110]^ A unique approach suggests inducing synthetic lethality in *Kras*-mutant tumors by targeting the oncogenic stress caused by *Kras* mutation. Stress granule formation in response to mutated *Kras* signaling is proposed as a therapeutic target.^[106]^ In our recent work, we highlight the crucial role of NUPR1 in stress granule formation due to *Kras*^*G12D*^ mutation-induced stress. NUPR1 inhibition with ZZW-115 prevents stress granule formation, supporting a potential therapeutic avenue.^[76]^ The findings propose targeting NUPR1-dependent stress granule formation as a promising strategy for mutated *Kras*^*G12D*^-associated tumors, presenting preclinical proof-of-concept for this approach. Our study explores why cells with stress granules are absent in nontransformed pancreatic areas, ruling out ZZW-115’s impact on *Kras*^*G12D*^ signaling. Instead, the formation of stress granules induced by NUPR1 overexpression is identified as crucial for *Kras*^*G12D*^ transformation. In vivo results support this, indicating a dependency of *Kras*^*G12D*^ cells on NUPR1 expression. The study’s significance lies in extending the understanding of pathophysiological mechanisms in *Kras*^*G12D*^-dependent cancer development. It suggests inhibiting NUPR1-dependent stress granule formation as a synthetic lethality therapeutic strategy for mutated *Kras*^*G12D*^-dependent tumors,^[76]^ providing valuable preclinical evidence.

### 19.1. Current challenges and future prospects

Despite the promise of targeting stress granules in cancer treatment, several challenges persist.^[104]^ A major challenge lies in the incomplete understanding of the molecular mechanisms that govern the formation and disassembly of stress granules. The precise cascade of events that drives their assembly and subsequent dissolution under different physiological or stress conditions remains to be fully elucidated. Additionally, the heterogeneity of stress granules across different cancer types complicates therapeutic targeting, as their role in cancer progression may vary depending on tumor context. Another major challenge is developing selective inhibitors that disrupt stress granule formation without affecting normal stress responses essential for cellular function. Since stress granules play a protective role under physiological stress conditions, broad inhibition could lead to unintended cytotoxicity in noncancerous cells. Therefore, identifying cancer-specific regulators of stress granule dynamics, such as NUPR1, is crucial for developing targeted therapies. Ongoing research is focused on elucidating the molecular mechanisms governing stress granule formation and identifying additional key regulators that could serve as therapeutic targets. Advances in single-cell analysis and high-resolution imaging techniques are helping to reveal the complexity of stress granules and their role in tumor progression. Additionally, combination therapies incorporating stress granule inhibitors with chemotherapy or immunotherapy are being explored to enhance treatment efficacy. Several preclinical studies are currently assessing the therapeutic potential of stress granule inhibitors in Kras-driven cancers.^[106]^ Preliminary findings suggest that targeting stress granules can sensitize tumors to chemotherapy and prevent therapy resistance. As research progresses, further refinement of stress granule-targeting strategies will be critical for their successful translation into clinical applications.

## 20. Discussion and conclusions

This review provides a panoramic and detailed overview of oncogenic stress, addressing both the molecular mechanisms that trigger malignant transformation (Fig. 1) and the adaptive responses that cells implement to survive this challenge (Fig. 2). Integrating these elements is crucial to understanding how, despite possessing robust control and repair systems, the aberrant activation of oncogenes, exemplified primarily by mutations in KRAS, can lead to cell cycle deregulation and tumor progression. To counter these effects, the cell activates multiple adaptive systems. HSPs serve as the first line of defense by facilitating proper protein folding and stabilizing oncoproteins that might otherwise lose functionality or form toxic aggregates. However, this same protective mechanism can favor the survival of malignant cells by sustaining the activity of proteins that drive tumor growth. The UPS and autophagy act complementarily to eliminate misfolded proteins and damaged organelles, thereby maintaining protein and metabolic homeostasis. Nonetheless, the overstimulation of these mechanisms in the oncogenic context can contribute to therapeutic resistance, as cancer cells become more efficient at removing harmful components. Another fundamental adaptive response is the activation of the NRF2-ARE pathway, which induces the expression of antioxidant enzymes to neutralize oxidative stress. Together with redox systems based on thioredoxin and glutathione, these pathways help restore oxidative balance, enabling cells to survive in environments that would normally be lethal. In parallel, the DDR, mediated by proteins such as ATM, ATR, and the tumor suppressor p53, attempts to repair the genomic instability induced by oncogene activation. However, the loss or mutation of p53 in many cancers reveals the vulnerability of this system when it is either overwhelmed by persistent stress or its function is compromised. Stress granules act as temporary reservoirs, regulating translation and protecting genetic material under adverse conditions. Their formation, which may be exacerbated in cells harboring KRAS mutations, is presented as a mechanism of resistance to chemotherapy and a potential therapeutic target. The possibility of inhibiting stress granule formation, for example, by modulating NUPR1, opens new avenues for targeting tumors that depend on these structures for survival. From a clinical perspective, this integrated understanding is particularly relevant. Identifying “therapeutic windows,” such as the dependency of tumors on autophagy, HSP activity, or stress granule formation, offers the possibility of designing synthetic lethality strategies. In other words, it may be possible to target the specific vulnerabilities that arise from oncogenic stress without critically affecting normal cells. Inhibiting key proteins in these processes, such as NUPR1 in the case of stress granules, represents a promising strategy to sensitize cancer cells to chemotherapy and other treatments.

In summary, the reviewed article offers a valuable integration of the mechanisms of oncogenic stress, linking oncogene activation with adaptive cellular responses and highlighting their clinical implications. The convergence of multiple signaling and response axes, from proliferative signaling to antioxidant and DNA repair responses, along with stress granule formation, illustrates the inherent complexity of cancer biology. This integrative approach not only enhances our understanding of the underlying pathological processes but also lays the groundwork for the development of targeted therapies that exploit the specific vulnerabilities of cancer cells. The critical discussion underscores the need for continued research into these interactions to design more effective and personalized therapeutic strategies in the fight against cancer.

## 21. Perspectives on targeting oncogenic stress: a new frontier in cancer therapy

Oncogenic stress is a double-edged sword in cancer progression, simultaneously triggering protective mechanisms and creating vulnerabilities that can be therapeutically exploited. While pathways such as HSPs, UPS, autophagy, and redox regulation help maintain cellular homeostasis, their dysregulation fosters tumor progression and therapy resistance. Understanding these processes opens new avenues for synthetic lethality strategies, multitarget therapies, and personalized medicine, offering hope for more effective and less toxic cancer treatments.

### 21.1. The complexity of oncogenic stress and its dual role:

The intricate balance between cellular survival and vulnerability under oncogenic stress reveals both protective and destructive forces at play. While HSPs, UPS, autophagy, and redox pathways help maintain cellular integrity, their overactivation paradoxically fosters tumor progression and therapy resistance. This insight highlights the need for precision-targeted therapies that selectively disrupt cancer-promoting adaptations without compromising normal cellular functions.

### 21.2. Synthetic lethality as a therapeutic opportunity

The concept of therapeutic windows is particularly compelling. Identifying cancer cell dependencies, such as heightened reliance on autophagy, stress granule formation, or redox regulation, paves the way for synthetic lethality strategies. For instance, inhibiting key proteins such as NUPR1 in stress granules or targeting the NRF2-ARE pathway in oxidative stress-prone tumors could selectively impair malignant cells while sparing normal ones.

### 21.3. The role of stress granules as emerging targets

The discussion underscores stress granules as a potential resistance mechanism in KRAS-mutant tumors, offering an underexplored therapeutic avenue. Modulating stress granule dynamics could disrupt mRNA translation control, protein homeostasis, and stress adaptation, ultimately sensitizing tumors to chemotherapy. The NUPR1 inhibition strategy could serve as a starting point for novel treatments that capitalize on stress granules’ essential role in oncogenic survival.

### 21.4. A systems biology approach to cancer treatment

Rather than viewing oncogenic stress responses as isolated pathways, this discussion highlights the interconnectedness of multiple survival mechanisms. From DNA repair (DDR) and redox regulation to protein homeostasis and metabolic adaptation, these systems act synergistically. Future therapeutic developments should consider multitarget strategies, potentially combining inhibitors of autophagy, stress responses, and antioxidant defenses to overcome tumor resilience.

### 21.5. The future of personalized medicine in cancer therapy

This review underscores the importance of patient-specific vulnerabilities in developing effective cancer therapies. With KRAS mutations and other oncogenic alterations shaping stress responses, future research should focus on identifying biomarkers that predict tumor dependencies. Personalized interventions targeting HSPs, DDR proteins, or stress granules could lead to more effective, less toxic treatment regimens tailored to individual tumor profiles.

## Author contributions

**Conceptualization:** Juan Iovanna, Martin E. Fernández Zapico, Jose Luis Neira, Patricia Santofimia-Castaño.

**Project administration:** Juan Iovanna.

**Supervision:** Juan Iovanna.

**Writing – original draft:** Juan Iovanna, Matías Estaras, Daniel Grasso, Martin E. Fernández Zapico, Jose Luis Neira, Patricia Santofimia-Castaño.

**Validation:** Matías Estaras, Daniel Grasso, Martin E. Fernández Zapico, Jose Luis Neira, Patricia Santofimia-Castaño.

**Visualization:** Matías Estaras, Daniel Grasso.
